# Comparing internet and face-to-face surveys as methods for eliciting preferences for social care-related quality of life: evidence from England using the ASCOT service user measure

**DOI:** 10.1007/s11136-019-02172-2

**Published:** 2019-04-03

**Authors:** Eirini-Christina Saloniki, Juliette Malley, Peter Burge, Hui Lu, Laurie Batchelder, Ismo Linnosmaa, Birgit Trukeschitz, Julien Forder

**Affiliations:** 1 Personal Social Services Research Unit, University of Kent, Canterbury, UK; 2 Centre for Health Services Studies, University of Kent, Canterbury, UK; 3 Personal Social Services Research Unit, London School of Economics, London, UK; 4 RAND Europe, Cambridge, UK; 5 Centre for Health and Social Economics, National Institute for Health and Welfare, Helsinki, Finland; 6 Department of Health and Social Management, University of Eastern Finland, Kuopio, Finland; 7 Research Institute for Economics of Aging, WU Vienna University of Economics and Business, Vienna, Austria

**Keywords:** Preferences, Face-to-face, Internet, Care, Quality of life, ASCOT

## Abstract

**Purpose:**

Traditionally, researchers have relied on eliciting preferences through face-to-face interviews. Recently, there has been a shift towards using internet-based methods. Different methods of data collection may be a source of variation in the results. In this study, we compare the preferences for the Adult Social Care Outcomes Toolkit (ASCOT) service user measure elicited using best–worst scaling (BWS) via a face-to-face interview and an online survey.

**Methods:**

Data were collected from a representative sample of the general population in England. The respondents (face-to-face: *n* = 500; online: *n* = 1001) completed a survey, which included the BWS experiment involving the ASCOT measure. Each respondent received eight best–worst scenarios and made four choices (best, second best, worst, second worst) in each scenario. Multinomial logit regressions were undertaken to analyse the data taking into account differences in the characteristics of the two samples and the repeated nature of the data.

**Results:**

We initially found a number of small significant differences in preferences between the two methods across all ASCOT domains. These differences were substantially reduced—from 15 to 5 out of 30 coefficients being different at the 5% level—and remained small in value after controlling for differences in observable and unobservable characteristics of the two samples.

**Conclusions:**

This comparison demonstrates that face-to-face and internet surveys may lead to fairly similar preferences for social care-related quality of life when differences in sample characteristics are controlled for. With or without a constant sampling frame, studies should carefully design the BWS exercise and provide similar levels of clarification to participants in each survey to minimise the amount of error variance in the choice process.

## Introduction

Measurement of the outcomes that people experience in using health and care services is a well-established and routinely used approach to assessing those services. This approach relies on robust and comprehensive outcome indicators that capture the relative preferences that people place on the range of ways that services can impact their quality of life. In long-term care—generally known as social care in the UK—little is known about relative preferences. Conventionally, preference studies have used face-to-face interviews with a paper-and-pencil or later with the assistance of a computer interface, with the interviewer being present. However, these are costly and time-consuming methods, which constrain how quickly the evidence-based practice can be developed. Recently, there has been a shift towards internet surveys to gather such data [1–4]. This method was used in a cross-national project to evaluate the impact of long-term care—the EXCELC project.^1^ It was also important to assess the robustness of internet-based approaches in comparison to face-to-face approaches; accordingly, part of the study used both methods.

Face-to-face (mail, paper- or computer-assisted) interviews can provide high-quality data with good completion rates and reliability, but as well as being expensive and time-consuming can also be limited to a certain geographical area, as compared to internet-based approaches [1, 2]. Internet-based surveys also make it possible to record response time accurately and target groups of respondents faster at lower cost [1]. However, with internet surveys it is difficult to achieve sample representativeness (of the general population), and data quality may be poor due to low engagement of the respondents or limited understanding of the questions [1, 3]. These advantages and disadvantages of the different methods may be a source of variation in the results, even if the questions are identical [5, 6].

The sources of variation in the results between the different administration methods can be twofold: (a) measurement effects and (b) sample composition (representation) effects [7]. The former concerns potential (intentional or self-deceptive) bias to give a socially desirable answer (social desirability bias) or putting insufficient effort towards answering the survey questions (satisficing). Whilst measurement effects relate to *how* someone responds, sample composition effects have more to do with *who* responds—for instance, the samples may be different between the administration methods due to differential non-response. Identifying a ‘pure’ method effect even if such effects are taken into account can prove challenging [7].

Several studies in environmental economics have compared preferences elicited from face-to-face interviews and internet surveys [1, 8, 9]. The studies used the discrete choice experiment (DCE) technique to elicit preferences and were heterogeneous in the sampling frame and environmental goods valued. No significant differences in preferences between the different administration methods were found, and the studies were unable to separate measurement from sample composition effects. However, the limited experimental control, different sampling frames and confounding of measurement with sample composition effects used in these studies could have driven the results [10]. Similar findings have been reported within health care research irrespective of the preference elicitation technique (adaptive conjoint analysis (ACA), person trade-off (PTO), DCE, time trade-off (TTO)), health-related outcomes and sampling process, with a randomised sampling being more common [2, 11, 12]. Adding to this evidence, Determann et al. [3] using the same sampling frame found that consumers’ preferences for health insurance were similar across a face-to-face (paper-based) and an online DCE. Finally, Norman et al. [13] found small differences in preferences for health-related quality of life across internet and face-to-face TTO tasks, but there were concerns over sample representativeness and small sample sizes that were not accounted for.

The EXCELC study used the best–worst scaling (BWS) technique—in contrast to techniques used in the above literature comparing survey methods—known for presenting one profile at a time and its arguably lower cognitive burden compared to a traditional DCE [14]. The main existing study for long-term (social) care research [15] also used BWS but with a face-to-face survey mode. Both that study and EXCELC elicited preferences for service users’ social care-related quality of life (SCRQoL) using the Adult Social Care Outcomes Toolkit (ASCOT)^2^ measure. ASCOT measures social care-related quality of life across eight domains: *accommodation cleanliness and comfort, safety, food and drink, personal care, control over daily life, social participation and involvement, dignity, occupation and employment*. Each domain has four levels, with higher levels indicating higher needs [15, 16]. ASCOT has been recommended for use in the economic evaluation of social care services [17–20].

To our knowledge, no study has examined whether preferences for service users’ SCRQoL differ across various administration methods. The specific aim of this paper is to compare preferences elicited from face-to-face and internet surveys for the BWS task using the ASCOT service user measure. For any differences in preferences identified between face-to-face and internet, we further seek to examine their causes particularly with respect to the sample composition effects between the two methods of data collection.^3^ This is part of a more general aim to establish relative preferences regarding care-related outcomes for people using long-term care in EXCELC.

## Methods

### Components of the surveys

Two surveys were undertaken during the same period: a face-to-face survey administered on laptops or tablets (i.e. administered through computer-assisted personal interview (CAPI)) and an online survey. Each survey included demographic questions to assist with the screening process, a brief introduction to the study and a consent form. Consenting participants in both surveys were asked a set of questions regarding: (a) their current quality of life (using the ASCOT measure), (b) imaginary situations where their circumstances have changed and their quality of life might be different (BWS exercise); (c) a follow-up about their understanding of the BWS exercise; (d) their experience of care and support; (e) further socio-economic and socio-demographic characteristics. The questions about socio-economic grade and region varied slightly between the two surveys, reflecting the different administration method. The face-to-face survey also included an extra set of questions relating to the interviewers’ assessment of how well the participant was able to undertake the BWS exercise,^4^ and the participants could ask for clarification throughout the interview. Both surveys were pilot tested, and the face-to-face survey has also been used in another study [15].

### Best–worst scaling experiment

The participants in both surveys were asked to put themselves in an imaginary situation where, through illness, accident or old age, they were not able to do everything they might expect to do for themselves without some assistance. To help picturing themselves in this situation, they were encouraged to think how they would go about all of their day-to-day activities, from getting up in the morning to going to bed at the end of the day.

**Fig. 1:**
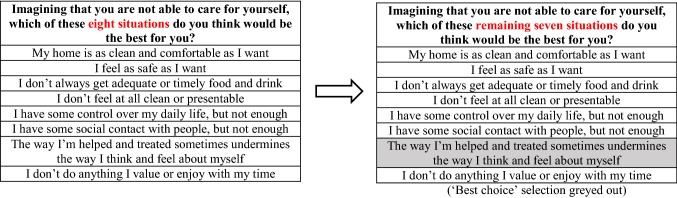
Best–worst scaling example for ASCOT service user measure

The respondents were then presented with a set of eight hypothetical scenarios. Each scenario contained eight attributes reflecting the eight ASCOT domains. Each attribute represented one out of four levels (1–4)—higher level indicated higher needs. Respondents were asked to select the best (or most preferred) choice from the scenario presented (‘profile’ case) [21] and the selected choice was then greyed out (see Fig. 1 for an illustration). The same process was followed for the worst (or least preferred) choice, the second best, and second worst choices. Therefore, each respondent made in total 32 choices (i.e. four choices in each of the eight scenarios). The order of the attributes was randomised between respondents to control for potential ordering biases [22, 23].

### Experimental design

The best–worst scaling scenarios were chosen using an orthogonal main effects plan. The full factorial plan consisted of 4^8^ possible profiles and was reduced to a design matrix of 32 scenarios. The design matrix was blocked further into four segments with each respondent receiving eight best–worst scenarios. This blocking procedure aimed to retain balance and minimise correlations within the blocks. A foldover design was used to eliminate ‘easy choices’ in each scenario [24].

### Data collection

Both surveys were conducted between June and July 2016; 1001 adults from the general population in England were recruited for the internet survey and 500 adults for the face-to-face survey. Sampling was targeted to be representative of the general population in England following quotas for key socio-demographic variables (age, gender, socio-economic grade and region).

The data collection for the face-to-face survey involved house-to-house recruitment following quotas for age (18–24, 25–34, 35–44, 45–54, 55–64, 65–79, 80 years or older), gender, socio-economic grade (A/B, C1, C2, D/E)^5^ and region (London, South East, Kent, West Midlands, North East). After completing the survey, the participants were rewarded with a £5 voucher and a thank you letter for their time and effort.

For the online survey, respondents were sourced from an online market research panel following quotas for age (18–24, 25–34, 35–44, 45–54, 55 years or older), gender and region (North East, North West, Yorkshire and the Humber, East Midlands, West Midlands, East of England, London, South East, South West) based on the general population in England. The respondents were invited to participate in the survey through an email invitation. The email invites were automatically randomised to members of the panel to minimise bias. The subject of the invitation was social research and a standard incentive appropriate to the length of the survey was offered when the respondent had completed the survey. The respondents had the option to withdraw from the survey at any time.

### Analysis

Respondent characteristics and time taken to complete the BWS exercise were compared across the face-to-face and online sample using Chi-square tests and independent *t*-tests.

We used a multinomial logit (MNL) regression model [25, 26] to estimate preferences for service users’ SCRQoL using ASCOT. The estimation process followed closely the study by Netten et al. [15]. Each attribute was specified as an alternative within the model, based on the random utility theory, with a utility function defined to take account of the level at which the attribute was presented within the scenario and the position of the attribute in the scenario (separately for the best and worst choices). The model assumed that all choices were independent and sequential.

The utility respondent *q* derived from choosing alternative *i* from a set of alternatives *J* was split into an explainable component *V*_*iq*_ and a random component *ε*_*iq*_,

1 $${U_{iq}}={V_{iq}}+{\varepsilon _{iq}},\forall J.$$

The random component ${\varepsilon _{iq}}\sim EV1$ (extreme value type 1) enabled choice data to be estimated using a closed form MNL as shown below:

2 $${P_{iq}}=\frac{{{{\text{e}}^{\theta {V_{iq}}}}}}{{\mathop \sum \nolimits_{{j \in J}} {{\text{e}}^{\theta {V_{jq}}}}}},$$

where ${P_{iq}}$ is the probability of each respondent *q* choosing alternative *i* from all relevant alternatives *j* in the choice set *J* and *θ* is the scale parameter which is inversely proportional to the standard deviation of the random component.

To evaluate differences in preferences for SCRQoL between the two methods of data collection, we considered estimating a pooled MNL model. In this model, the utility function for a given attribute was (a) a linear-additive function of the products between the coefficients (preference weights) to be estimated, the dummy-coded attribute levels (with only one level at a time taking the value of 1 for a given choice) and dataset type (face-to-face (f2f) or internet); and (b) a set of dummy-coded variables to control for the position of the attribute level in the scenario when that attribute was chosen as being best, worst, second best or second worst. Effects coding was used to dissociate best and worst choices. The results from a formal pooling test introduced by Swait and Louviere [27] confirmed that it is appropriate to combine the two datasets as long as differences in scale variance are taken into account.^6^ Failing to account for scale heterogeneity would lead to biased results [28]. We first estimated a pooled model that controlled for scale differences between the two datasets.

A generalised example of the utility function specification for the safety attribute in the pooled model is shown below:

3 $$\begin{aligned} {U_q}({\text{safety}})= & {\alpha _1}{\text{*}}{\left( {1,\;{\text{if}}\;{\text{safety}}\;{\text{level}}=1} \right)_{\text{i}}}{\text{*}}{\left( {1,\;{\text{if}}\;{\text{choice}}={\text{best}}\;{\text{or}}\;{\text{second}}\;{\text{best}}} \right)_{\text{i}}}{\text{*}}(1,\;{\text{if}}\;{\text{f}}2{\text{f}}\;{\text{data}}) \\ & \quad - {\alpha _1}{\text{*}}{\left( {1,\;{\text{if}}\;{\text{safety}}\;{\text{level}}=1} \right)_{\text{i}}}{\text{*}}{\left( {1,\;{\text{if}}\;{\text{choice}}={\text{worst}}\;{\text{or}}\;{\text{second}}\;{\text{worst}}} \right)_{\text{i}}}{\text{*}}(1,\;{\text{if}}\;{\text{f}}2{\text{f}}\;{\text{data}}) \\ & \quad \quad \quad \quad \quad \quad \quad \quad \quad \quad \quad \quad \quad \quad \quad \quad \quad \quad\vdots \\ & \quad +{\alpha _4}{\text{*}}{\left( {1,\;{\text{if}}\;{\text{safety}}\;{\text{level}}=4} \right)_{\text{i}}}{\text{*}}{\left( {1,\;{\text{if}}\;{\text{choice}}={\text{best}}\;{\text{or}}\;{\text{second}}\;{\text{best}}} \right)_{\text{i}}}{\text{*}}(1,\;{\text{if}}\;{\text{f}}2{\text{f}}\;{\text{data}}) \\ & \quad - {\alpha _4}{\text{*}}{\left( {1,\;{\text{if}}\;{\text{safety}}\;{\text{level}}=4} \right)_{\text{i}}}{\text{*}}{\left( {1,\;{\text{if}}\;{\text{choice}}={\text{worst}}\;{\text{or}}\;{\text{second}}\;{\text{worst}}} \right)_{\text{i}}}{\text{*}}(1,\;{\text{if}}\;{\text{f}}2{\text{f}}\;{\text{data}}) \\ & \quad +{\beta _1}{\text{*}}{\left( {1,\;{\text{if}}\;{\text{safety}}\;{\text{level}}=1} \right)_{\text{i}}}{\text{*}}{\left( {1,\;{\text{if}}\;{\text{choice}}={\text{best}}\;{\text{or}}\;{\text{second}}\;{\text{best}}} \right)_{\text{i}}}{\text{*}}(1,\;{\text{if}}\;{\text{internet}}\;{\text{data}}) \\ & \quad - {\beta _1}{\text{*}}{\left( {1,\;{\text{if}}\;{\text{safety}}\;{\text{level}}=1} \right)_{\text{i}}}{\text{*}}{\left( {1,\;{\text{if}}\;{\text{choice}}={\text{worst}}\;{\text{or}}\;{\text{second}}\;{\text{worst}}} \right)_{\text{i}}}{\text{*}}(1,\;{\text{if}}\;{\text{internet}}\;{\text{data}}) \\ & \quad \quad \quad \quad \quad \quad \quad \quad \quad \quad \quad \quad \quad \quad\quad\quad\quad \vdots \\ & \quad +{\beta _4}{\text{*}}{\left( {1,\;{\text{if}}\;{\text{safety}}\;{\text{level}}=4} \right)_i}{\text{*}}{\left( {1,\;{\text{if}}\;{\text{choice}}={\text{best}}\;{\text{or}}\;{\text{second}}\;{\text{best}}} \right)_i}{\text{*}}(1,\;{\text{if}}\;{\text{internet}}\;{\text{data}}) \\ & \quad - {\beta _4}{\text{*}}{\left( {1,\;{\text{if}}\;{\text{safety}}\;{\text{level}}=4} \right)_i}{\text{*}}{\left( {1,\;{\text{if}}\;{\text{choice}}={\text{worst}}\;{\text{or}}\;{\text{second}}\;{\text{worst}}} \right)_i}{\text{*}}(1,\;{\text{if}}\;{\text{internet}}\;{\text{data}}) \\ & \quad +{\gamma _1}{\text{*}}{\left( {1,\;{\text{if}}\;{\text{safety}}\;{\text{appeared}}\;{\text{in}}\;{\text{first}}\;{\text{row}}} \right)_i}{\text{*}}{(1,\;{\text{if}}\;{\text{choice}}={\text{best}}\;{\text{or}}\;{\text{second}}\;{\text{best}})_i} \\ & \quad \quad \quad \quad \quad \quad \quad \quad \quad \quad \quad \quad \quad \quad\quad\quad \quad \vdots \\ & \quad +{\gamma _8}{\text{*}}{\left( {1,\;{\text{if}}\;{\text{safety}}\;{\text{appeared}}\;{\text{in}}\;{\text{eighth}}\;{\text{row}}} \right)_i}{\text{*}}{(1,\;{\text{if}}\;{\text{choice}}={\text{best}}\;{\text{or}}\;{\text{second}}\;{\text{best}})_i} \\ & \quad - {\delta _1}{\text{*}}{\left( {1,\;{\text{if}}\;{\text{safety}}\;{\text{appeared}}\;{\text{in}}\;{\text{first}}\;{\text{row}}} \right)_i}{\text{*}}{(1,\;{\text{if}}\;{\text{choice}}={\text{worst}}\;{\text{or}}\;{\text{second}}\;{\text{worst}})_i} \\ & \quad \quad \quad \quad \quad \quad \quad \quad \quad \quad \quad \quad \quad \quad \quad\quad\quad\vdots \\ & \quad - {\delta _8}{\text{*}}{\left( {1,\;{\text{if}}\;{\text{safety}}\;{\text{appeared}}\;{\text{in}}\;{\text{eighth}}\;{\text{row}}} \right)_i}{\text{*}}{(1,\;{\text{if}}\;{\text{choice}}={\text{worst}}\;{\text{or}}\;{\text{second}}\;{\text{worst}})_i} \\ & \quad +{\varepsilon _{\text{i}}}, \\ \end{aligned} $$

where $${\alpha _1}, \ldots ,{\alpha _4}$$$$({\beta _1}, \ldots ,{\beta _4})$$ are the coefficients for each attribute level in the face-to-face (internet) dataset; $${\gamma _1}, \ldots ,~{\gamma _8}$$$$({\delta _1}, \ldots ,~{\delta _8})$$ are the coefficients for the position of the safety attribute within the best–worst scenario if the choice was best or second best (worst or second worst);* ε*_*i*_ is the error term.

The coefficients were estimated separately for each dataset within the pooled model except for the position coefficients and those coefficients for the control attribute at level 1 and level 4. The latter coefficients were jointly estimated across the two datasets. Moreover, the attribute control over daily life at level 4 was used as a reference level and was set to zero [15]. To avoid over-identification, the position coefficients of the first attribute in the scenario for the best and worst choices as well as the constant were also set to zero. The model also included a scale parameter to allow for the possibility that one of the datasets might have higher error variance than the other.

Controlling for taste heterogeneity in the model is important, particularly if there are significant differences in the sample composition of the two datasets. Therefore, an additional pooled model controlled for differences in scale heterogeneity between datasets or different groups of respondents across the two datatsets, and shared taste heterogeneity across the two datasets [15] .^7^ The additional scale parameters were jointly estimated in the model including best or worst choice, education and time taken to complete the BWS exercise. For instance, we used the first quartile and the median to calculate a binary indicator of fast-long BWS completion time, in case faster completion of the BWS exercise was indicative of low engagement of the online respondents compared to those who had an interviewer present. Common terms were used across the two datasets to capture the impact of socio-economic and socio-demographic factors (taste heterogeneity).^8^

The MNL models were estimated first using ALOGIT [29]. To account for the repeated nature of the data, robust standard errors were subsequently obtained using the sandwich estimator [15] and were estimated in BIOGEME [30].^9^

## Results

Each respondent in the face-to-face (*n* = 500) and internet survey (*n* = 1001) made 32 choices—a total sample of 16000 observations for face-to-face and 32032 observations for internet. The full sample was used in the pooled model (48032 observations), but was reduced to 47296 observations when controlling for taste and scale heterogeneity due to a number of individuals not disclosing their education (0.8% for face-to-face and 1.9% for internet), one of the scale parameters in the model.

### Sample characteristics

Descriptive statistics of the main socio-demographic and socio-economic variables are reported in Table 1. The majority of face-to-face participants (80%) did not have a degree, and were significantly less educated than the internet respondents—about 40% of the internet respondents had at least a degree. The internet sample comprised fewer semi or unskilled manual workers (17% versus 28%), but more individuals in higher managerial or administrative positions (44% vs. 23%) than those in the face-to-face sample. In comparison to the general population, both internet and face-to-face samples differed in terms of the top and bottom education categories (below secondary education, and degree and above, respectively). Both samples also included a larger number of retired individuals and individuals aged between 55 and 64, but fewer skilled or manual workers compared to the general population. The internet and face-to-face samples were very similar to the general population in terms of gender.

**Table 1:** Descriptive statistics

	Face-to-face	Internet	General population	*p-*value (face-to-face vs. internet)^i^
*n*	500	1001	Varies	
Gender^a^ (%)				0.570
Male	46.2	47.8	48.6	
Female	53.8	52.2	51.4	
Age category^b^ (years) (%)				0.246
18–24	12.0	9.6	11.4	
25–34	16.8	17.5	17.4	
35–44	14.6	16.8	16.5	
45–54	18.0	18.3	17.9	
55–64	16.2	19.1	14.3	
65+	22.4	18.8	22.5	
Education^c^ (%)				< 0.001
Below secondary education	17.6	3.9	35.8	
Lower secondary and upper secondary	50.4	43.9	15.2	
Short-cycle tertiary and post-secondary	11.6	11.1	15.9	
Degree and above (BA/MA/PhD or equivalent)	19.6	39.3	27.4	
Don’t know/other	0.8	1.9	5.7	
Tenure^d^ (%)				< 0.001
Own house or apartment	51.4	72.7	64.1	
Rent	44.4	23.1	34.5	
Other	3.6	4.2	1.3	
Missing	0.6	0.0	–	
Current employment status^e^ (%)				0.133
In full- or part-time paid work	54.8	59.3	62.1	
In education, even if on vacation	4.8	5.0	9.2	
Unemployed-actively or not actively looking for a job	4.8	3.1	4.4	
Permanently sick or disabled	5.0	2.6	4.1	
Retired	23.6	23.7	13.7	
In community/military service/doing housework, looking after children or other persons	5.8	5.3	4.4	
Don’t know/other	1.2	1.0	2.2	
Religion^f^ (%)				< 0.001
No religion	35.0	45.9	23.4	
Christian^g^	57.2	46.2	61.7	
Buddhist/Hindu/Jewish/Muslim/Sikh	6.8	4.7	7.4	
Any other religion	0.6	1.1	0.5	
Prefer not to say	0.4	2.2	7.0	
Social grade^h^ (%)				< 0.001
A/B	23.0	43.9	22.9	
C1	30.4	28.2	30.4	
C2	18.2	10.2	21.9	
D/E	28.2	16.9	24.8	
Other	0.2	0.9	–	

Descriptive statistics are reported for those variables with available general population estimates

^a^*Source* 2011 Census for England, population estimates for those aged 18+ (https://www.nomisweb.co.uk/census/2011/DC1117EW/view/2092957699?rows=c_age&cols=c_sex, Last accessed 25/07/2018)

^b^*Source* 2015 Analysis Tool, Office for National Statistics (https://www.ons.gov.uk/peoplepopulationandcommunity/populationandmigration/populationestimates, Last accessed: 25/07/2018)

^c^*Source* 2011 Census for England, population estimates for those aged 16+ (https://www.nomisweb.co.uk/census/2011/DC5107EWLA/view/2092957699?rows=c_hlqpuk11&cols=c_age, Last accessed: 25/07/2018)

^d^*Source* 2011 Census for England, all residents (https://www.nomisweb.co.uk/census/2011/QS403EW/view/2092957699?rows=rural_urban&cols=c_tenhuk11, Last accessed: 25/07/2018)

^e^*Source* 2011 Census for England, population estimates for those aged 16 to 74 (https://www.nomisweb.co.uk/census/2011/KS601EW/view/2092957699?rows=cell&cols=c_sex, Last accessed: 25/07/2018)

^f^Source: 2011 Census for England, population estimates for those aged 18+ (https://www.nomisweb.co.uk/census/2011/DC2107EW/view/2092957699?rows=c_age&cols=c_relpuk11, Last accessed: 25/07/2018)

^g^Christian includes Church of England, catholic, protestant and all other Christian denominations

^h^*Source* 2011 Census for England, population estimates for those aged 16 to 64 (https://www.nomisweb.co.uk/census/2011/QS613EW/view/2092957699?cols=measures, Last accessed: 25/07/2018)

^i^These are based on Chi-square tests

The time taken to complete the BWS exercise for each sample is reported in Table 2. Respondents in the internet sample completed the BWS exercise significantly faster than the face-to-face sample; median duration of 8.5 min (IQR 8.4–14.6) versus 11.0 min (IQR 6.5–11.9), respectively.^10^ Further descriptive statistics on the participant’s assessment of the BWS exercise are also shown in Table 2. Overall, over half of the participants in either of the surveys could all of the time put themselves into the imaginary situations described in the BWS exercise, understood the situations presented to them, and found them fairly easy to complete—which further support the argument of lower cognitive burden imposed by the BWS technique [14]. Despite this positive assessment of the BWS exercise by the participants, the internet and face-to-face samples differed substantially in the top categories of these questions, which may have been driven by the presence of the interviewer in the face-to-face sample. In particular, a larger percentage of the face-to-face respondents (67%) could put themselves in the imaginary situations described in the BWS exercise than those in internet (51%). Similarly, slightly over 30% of the face-to-face sample found the BWS exercise very easy to complete compared to just 11% of the internet sample.

**Table 2:** Descriptive statistics relating to the BWS exercise

	Face-to-face	Internet	*p-*value (face-to-face vs. internet)^c^
*n*	500	1001	
Time taken to complete the BWS exercise (min), median	11.0	8.5	< 0.001
(IQR)^a^	(8.4–14.6)	(6.5–11.9)	
Respondent’s assessment			
Put yourself in imaginary situations described in the BWS (%)			< 0.001
Yes, all of the time	67.0	51.0	
Yes, but only some of the time	28.8	42.7	
No	4.2	6.4	
Understood the situations in the BWS^b^ (%)			< 0.001
Yes, all of the time	89.4	75.8	
Yes, but only some of the time	10.0	22.0	
No	0.6	2.2	
How easy or difficult to complete the BWS (%)			< 0.001
Very easy	32.4	10.9	
Fairly easy	51.0	59.3	
Fairly difficult	14.4	27.6	
Very difficult	2.2	2.2	

*IQR* interquartile range

^a^We report the median and interquartile range due to a number of outliers (respondents left the survey open in their browser for a long time) in the internet sample

^b^The wording of the responses was slightly different in the face-to-face survey: “Yes, all of them”, “Yes, but only some of them”, “No”

^c^These are based on an independent *t*-test for the time taken to complete the BWS exercise and Chi-square tests for the variables relating to the understanding of the BWS exercise

### Model results

Table 3 shows the coefficients from the pooled model. It additionally reports whether the differences between these coefficients across the two datasets are statistically significant. From independent *t*-tests [31], we can see that half of the coefficient differences compared are statistically significant at the 5% level. The remaining coefficient differences are not statistically significant. Interestingly, respondents who completed the face-to-face survey placed lower values on the top two levels and higher values on the bottom level of almost all attributes compared to those who completed the internet survey. The only exception was the *social participation and involvement* attribute. The scale parameter on the dataset type is below one, and statistically different from the one at the 0.1% level, suggesting that there is higher error variance in the internet dataset than the face-to-face dataset.

**Table 3:** Pooled model

Attribute level	Face-to-face	Internet	Difference (face-to-face vs. internet)
Accommodation cleanliness and comfort			
1. My home is as clean and comfortable as I want	3.001 (0.110)	3.169 (0.115)	− 0.168 (0.113)
2. My home is adequately clean and comfortable	2.696 (0.100)	2.845 (0.108)	− 0.149 (0.104)
3. My home is not quite clean or comfortable enough	1.465 (0.081)	1.510 (0.070)	− 0.045 (0.076)
4. My home is not at all clean or comfortable	1.120 (0.074)	1.031 (0.059)	0.090 (0.067)
Safety			
1. I feel as safe as I want	3.191 (0.122)	3.256 (0.122)	− 0.065 (0.122)
2. Generally I feel adequately safe, but not as safe as I would like	1.675 (0.088)	1.749 (0.075)	− 0.074 (0.082)
3. I feel less than adequately safe	1.065 (0.077)	0.901 (0.061)	0.164 (0.070)*
4. I don’t feel at all safe	0.438 (0.072)	0.232 (0.059)	0.206 (0.066)**
Food and drink			
1. I get all the food and drink I like when I want	3.276 (0.114)	3.556 (0.130)	− 0.280 (0.123)*
2. I get adequate food and drink at OK times	2.894 (0.104)	2.974 (0.112)	− 0.080 (0.109)
3. I don’t always get adequate or timely food and drink	0.870 (0.072)	0.757 (0.062)	0.112 (0.067)
4. I don’t always get adequate or timely food and drink, and I think there is a risk to my health	0.616 (0.074)	0.207 (0.062)	0.409 (0.068)***
Personal care			
1. I feel clean and am able to present myself the way I like	3.314 (0.122)	3.477 (0.129)	− 0.163 (0.126)
2. I feel adequately clean and presentable	2.855 (0.111)	3.124 (0.118)	− 0.268 (0.114)*
3. I feel less than adequately clean or presentable	1.034 (0.082)	0.793 (0.063)	0.240 (0.073)**
4. I don’t feel at all clean or presentable	0.629 (0.072)	0.562 (0.061)	0.067 (0.067)
Control over daily life			
1. I have as much control over my life as I want	4.016 (0.131)		n/a
2. I have adequate control over my daily life	3.513 (0.122)	3.786 (0.135)	− 0.273 (0.129)*
3. I have some control over my daily life, but not enough	1.848 (0.101)	2.063 (0.086)	− 0.215 (0.094)*
4. I have no control over my daily life	0.000 (0.000)		n/a
Social participation and involvement			
1. I have as much social contact as I want with people I like	3.396 (0.124)	3.302 (0.120)	0.094 (0.122)
2. I have adequate social contact with people	2.879 (0.109)	2.879 (0.107)	0.001 (0.108)
3. I have some social contact with people, but not enough	1.721 (0.091)	1.913 (0.080)	− 0.192 (0.086)*
4. I have little social contact with people and feel socially isolated	0.742 (0.071)	0.808 (0.061)	− 0.066 (0.066)
Dignity			
1. The way I’m helped and treated makes me think and feel better about myself	3.480 (0.126)	3.776 (0.138)	− 0.295 (0.132)*
2. The way I’m helped and treated does not affect the way I think or feel about myself	2.631 (0.109)	2.671 (0.103)	− 0.039 (0.106)
3. The way I’m helped and treated sometimes undermines the way I think and feel about myself	1.032 (0.079)	0.987 (0.067)	0.045 (0.073)
4. The way I’m helped and treated completely undermines the way I think and feel about myself	0.803 (0.078)	0.539 (0.061)	0.264 (0.070)***
Occupation and employment			
1. I’m able to spend my time as I want, doing things I value or enjoy	3.699 (0.132)	4.156 (0.148)	− 0.458 (0.140)**
2. I’m able to do enough of the things I value or enjoy with my time	3.501 (0.124)	4.005 (0.144)	− 0.504 (0.134)***
3. I do some of the things I value or enjoy with my time, but not enough	2.144 (0.097)	2.333 (0.093)	− 0.189 (0.095)*
4. I don’t do anything I value or enjoy with my time	0.643 (0.067)	0.375 (0.057)	0.268 (0.062)***
Domain position effects by choice type	Yes	Yes	n/a
Scale parameters^a^			
Dataset type: internet	0.870 (0.037)***		
Model diagnostics			
No. of observations	48032		
df	76		
Final log-likelihood	− 71423.3		
Rho-squared(0)	0.199		
AIC	142999		

©University of Kent: The ASCOT measure is reproduced with permission from the University of Kent. All rights reserved

Robust standard errors are reported in parentheses. The significance of the differences has been evaluated using a variant of independent *t*-test

***p < 0.001, **p < 0.01, *p < 0.05; Robust standard errors in parentheses

^a^*Base* Dataset type: face-to-face

After controlling for observed differences in sample composition using taste and additional (unobserved) scale heterogeneity, the number of significant differences between the internet and face-to-face coefficients reduces substantially five out of the 30 coefficient differences compared which are statistically significant at the 5% level (see Table 4 for more details). Most highly significant differences are at level 4 of *food and drink, dignity* and *occupation and employment* attributes and at level 3 of the *personal care* attribute. The remaining significant coefficient difference is at level 4 of the *safety* attribute. All (five) significant differences were positive indicating that respondents in the face-to-face data placed higher value on the levels of these attributes compared to those completing the internet survey, and remained low in absolute value. It is important to note that these results do not directly compare to the results in Table 3. This is due to the reduced sample in the taste and scale heterogeneity model because of individuals not disclosing their education, and education being chosen as one of the scale parameters. All scale parameters (including the one on the dataset type) in this model are strongly statistically different from one—participants with education below degree made less deterministic choices compared to those with degree and above. Similarly, there was higher error variance (i.e. lower certainty) when participants made their worst choices or if they completed the BWS exercise face-to-face in less than 8 min or online in less than 7 min.

**Table 4:** Pooled model with scale and taste heterogeneity

Attribute level^a^	Face-to-face	Internet	Difference (face-to-face vs. internet)
Accommodation cleanliness and comfort			
1. My home is as clean and comfortable as I want	2.826 (0.180)	2.888 (0.183)	− 0.063 (0.182)
2. My home is adequately clean and comfortable	2.506 (0.164)	2.570 (0.166)	− 0.064 (0.165)
3. My home is not quite clean or comfortable enough	1.069 (0.096)	1.068 (0.087)	0.001 (0.092)
4. My home is not at all clean or comfortable	0.698 (0.079)	0.567 (0.063)	0.131 (0.072)
All levels: respondent has degree education and above	− 0.256 (0.046)		n/a
Safety			
1. I feel as safe as I want-annual household income £21,690 or above (Deciles 5–10)	2.836 (0.189)	2.824 (0.180)	0.013 (0.185)
1. I feel as safe as I want-annual household below £21,690 (Deciles 1–4)	3.035 (0.201)		n/a
2. Generally I feel adequately safe, but not as safe as I would like—respondent has below degree education	1.365 (0.116)	1.376 (0.107)	− 0.011 (0.112)
2. Generally I feel adequately safe, but not as safe as I would like—respondent has a degree or higher education	1.372 (0.101)		n/a
3. I feel less than adequately safe—respondent has quality of life “Very good” or “So good, it could not be better”	0.585 (0.092)	0.517 (0.080)	0.068 (0.086)
3. I feel less than adequately safe—respondent has quality of life “Good” or “Alright” or “Bad” or “Very bad” or “So bad, it could not be worse”	0.628 (0.070)		n/a
4. I don’t feel at all safe	0.030 (0.070)	− 0.131 (0.059)	0.161 (0.065)*
All levels: respondent is aged below 45	0.275 (0.046)		n/a
All levels: respondent has no experience with long-term needs	− 0.151 (0.048)		n/a
Food and drink			
1. I get all the food and drink I like when I want	3.046 (0.190)	3.131 (0.197)	− 0.085 (0.194)
2. I get adequate food and drink at OK times	2.661 (0.166)	2.605 (0.165)	0.056 (0.166)
3. I don’t always get adequate or timely food and drink	0.502 (0.075)	0.378 (0.061)	0.124 (0.068)
4. I don’t always get adequate or timely food and drink, and I think there is a risk to my health	0.203 (0.075)	− 0.179 (0.060)	0.383 (0.068)***
All levels: three or more adults in household	0.158 (0.051)		n/a
Personal care			
1. I feel clean and am able to present myself the way I like—respondent is male	3.068 (0.203)	2.893 (0.188)	0.175 (0.196)
1. I feel clean and am able to present myself the way I like—respondent is female	3.207 (0.199)		n/a
2. I feel adequately clean and presentable	2.643 (0.170)	2.758 (0.172)	− 0.115 (0.171)
3. I feel less than adequately clean or presentable	0.646 (0.087)	0.412 (0.064)	0.234 (0.076)**
4. I don’t feel at all clean or presentable	0.231 (0.071)	0.193 (0.061)	0.039 (0.066)
All levels: respondent is aged 18–24	− 0.186 (0.072)		n/a
All levels: respondent is aged 65 and above	0.227 (0.060)		n/a
Control over daily life			
1. I have as much control over my life as I want—respondent has a religion	3.163 (0.198)		n/a
1. I have as much control over my life as I want—respondent does not have a religion	3.316 (0.210)		n/a
2. I have adequate control over my daily life—respondent is not aged 55–64	2.846 (0.180)	2.929 (0.185)	− 0.084 (0.183)
2. I have adequate control over my daily life—respondent is aged 55–64	3.132 (0.204)		n/a
3. I have some control over my daily life, but not enough—household with either one adult or three or more adults	1.561 (0.128)	1.710 (0.120)	− 0.149 (0.124)
3. I have some control over my daily life, but not enough—household with two adults	1.627 (0.110)		n/a
4. I have no control over my daily life	0.000 (0.000)	0.000 (0.000)	n/a
All levels: respondent reports “I feel as safe as I want”/“Generally I feel adequately safe, but not as safe as I would like”	0.526 (0.042)		n/a
All levels: respondent rents house/apartment	− 0.306 (0.101)		n/a
Social participation and involvement			
1. I have as much social contact as I want with people I like	3.172 (0.204)	2.945 (0.187)	0.227 (0.196)
2. I have adequate social contact with people	2.661 (0.172)	2.529 (0.161)	0.132 (0.167)
3. I have some social contact with people, but not enough—respondent has a religion	1.426 (0.131)	1.588 (0.118)	− 0.163 (0.125)
3. I have some social contact with people, but not enough—respondent does not have a religion	1.479 (0.106)		n/a
4. I have little social contact with people and feel socially isolated	0.343 (0.077)	0.407 (0.061)	− 0.064 (0.069)
All levels: respondent is aged below 45	0.097 (0.043)		n/a
All levels: respondent reports “I do some of the things I value or enjoy with my time, but not enough / I don’t do anything I value or enjoy with my time”	− 0.166 (0.062)		n/a
Dignity			
1. The way I’m helped and treated makes me think and feel better about myself—respondent has quality of life “Good” or “Alright”	3.084 (0.207)	3.099 (0.199)	− 0.015 (0.203)
1. The way I’m helped and treated makes me think and feel better about myself—respondent has quality of life “So good, it could not be better” or “Very good” or “Bad” or “Very bad” or “So bad, it could not be worse”	3.233 (0.199)		n/a
2. The way I’m helped and treated does not affect the way I think or feel about myself—respondent has below degree education	2.346 (0.154)	2.191 (0.144)	0.155 (0.149)
2. The way I’m helped and treated does not affect the way I think or feel about myself—respondent has a degree or higher education	2.256 (0.147)		n/a
3. The way I’m helped and treated sometimes undermines the way I think and feel about myself	0.759 (0.085)	0.724 (0.070)	0.035 (0.078)
4. The way I’m helped and treated completely undermines the way I think and feel about myself	0.525 (0.081)	0.299 (0.060)	0.226 (0.071)**
All levels: respondent reports “I do some of the things I value or enjoy with my time, but not enough / I don’t do anything I value or enjoy with my time”	0.127 (0.063)		n/a
All levels: respondent has experience with long-term care needs	0.198 (0.047)		n/a
All levels: respondent is female	0.141 (0.046)		n/a
Occupation and employment			
1. I’m able to spend my time as I want, doing things I value or enjoy—respondent is not retired	3.105 (0.195)	3.274 (0.210)	− 0.169 (0.203)
1. I’m able to spend my time as I want, doing things I value or enjoy—respondent is retired	3.363 (0.218)		n/a
2. I’m able to do enough of the things I value or enjoy with my time—respondent is aged below 65	2.934 (0.186)	3.138 (0.200)	− 0.205 (0.193)
2. I’m able to do enough of the things I value or enjoy with my time—respondent is aged 65 and above	3.185 (0.207)		n/a
3. I do some of the things I value or enjoy with my time, but not enough	1.814 (0.125)	1.885 (0.120)	− 0.071 (0.123)
4. I don’t do anything I value or enjoy with my time	0.532 (0.068)	0.304 (0.054)	0.228 (0.061)***
All levels: respondent does not receive disability benefits	0.280 (0.047)		n/a
All levels: respondent has degree education and above	0.166 (0.046)		n/a
All levels: respondent has experience with long-term care needs	0.114 (0.041)		n/a
All levels: respondent is male	0.129 (0.045)		n/a
Domain position effects by choice type	Yes	Yes	n/a
Scale parameters^b^			
Dataset type: internet	0.905 (0.037)**		
BWS exercise completion time: duration ≥ 8.4 min (504 s) for face-to-face; duration ≥ 6.5 min (389 s) for internet	1.295 (0.060)***		
Education: below degree	0.830 (0.034)***		
Choice: worst or second worst	0.915 (0.019)***		
Model diagnostics			
No. of observations	47296		
df	109		
Final log-likelihood	− 69371.5		
Rho-squared(0)	0.210		
AIC	138961		

These values reflect the lower quartile in each dataset

Robust standard errors are reported in parentheses. The significance of the differences has been evaluated using a variant of independent *t*-test

©University of Kent: The ASCOT measure is reproduced with permission from the University of Kent. All rights reserved

***p < 0.001, **p < 0.01, *p < 0.05; Robust standard errors in parentheses

^a^The coefficients on the overall domain have been estimated irrespective of dataset type

^b^*Base* Dataset type: face-to-face; BWS exercise completion time: duration < 8.4 min (504 s) for face-to-face and < 6.5 min (389 s) for internet based on the lower interquartile; Education: degree and above; Choice: best or second best

## Discussion

Different administration methods have been used in the literature to elicit preferences; from paper-or computer-based face-to-face interviews to online surveys. Each method has its advantages and disadvantages with potential effects on the quality of the data and estimation of results, even if the questionnaires are identical. In this paper, we examined whether there are differences in preferences for service users’ SCRQoL elicited using BWS from a face-to-face (CAPI) and an internet survey. For any identified differences in preferences between the two methods, we further investigated whether they could be explained by differences in the characteristics of the two samples. We found a number of significant differences in preferences for SCRQoL using ASCOT between the face-to-face and internet samples across all attributes. However, given the large number of coefficient differences tested and them not being large in value (in absolute terms), we would expect some to appear significant by chance.

Our samples differed significantly in terms of key (observable) socio-demographic characteristics such as age, education and social grade, but were broadly representative of the general population. For example, and consistent with the literature, there was a significantly higher proportion of older respondents (aged 65+) in the face-to-face sample than the online sample [2, 12]. In addition, significantly fewer respondents in the face-to-face sample had a degree or further education compared to those online, but both samples underrepresented individuals with below secondary education in the general population which is consistent with a recent study by Liu et al. [32].

After controlling for taste and scale heterogeneity—to account for the differences in sample composition—the number of significant differences in preferences for SCRQoL between the two administration methods reduced substantially to five and the size of the difference was relatively small. The majority of significant differences were at level 4 of the respective attributes—these attributes were among the least frequently chosen as best choices, but most frequently chosen as worst choices in either samples. Finding slightly more error variance in the worst choices than the best choices is consistent with framing effects in the stated preferences literature [15].

Differences in unobservable characteristics between the two samples or more broadly scale effects would relate to variations in the levels of certainty (and potential error) with which different samples or groups of respondents express their preferences [9, 15, 27]. Internet respondents spent significantly less time completing the BWS exercise compared to their face-to-face counterparts—this may be a result of online participants being drawn from a panel, and thus being more familiar with this type of exercise. Faster completion time of the BWS exercise may also raise a question about the level of engagement of the online participants [2]. Given the broadly positive responses to the questions relating to the understanding of the BWS exercise, we do not consider engagement a significant problem in this study.

We should acknowledge that this study is not without limitations. First, although we accounted for a number of scale factors, there were unobserved characteristics that we were unable to control for. These relate, but are not exclusive, to the cognitive ability of the participants in either samples, the completion of the online survey with the help from or by someone else other than the targeted individual, learning and fatigue effects [33] and measurement error effects. Second, in this study, we did not use the same sampling frame in the way of asking the same people to fill in both surveys or by drawing participants with the exact same characteristics—this could reduce further the observed sample composition effects. However, we tried to account for observed sample composition effects by controlling for a number of taste heterogeneity factors. Overall, it appears that the method effects were small, implying that we can be sufficiently confident in the internet results, at least from a practical standpoint. Nonetheless, if further unobserved differences in the sample exist, then the small differences in preferences that we observe may not be ‘pure’ method effects.

Future BWS studies may want to draw respondents from the same randomised sample in both internet and face-to-face surveys to eliminate any differences in observable characteristics between the two samples—this may, however, come at a higher cost. Even if the two samples are the same, it is necessary to provide a similar level of clarification in the online survey as would be provided in the face-to-face survey. This could increase the survey understanding and certainty of choices of the online respondents with potential effects on the identification of significant differences between the different administration methods. For example, future BWS studies may want to include video explanations prior to the presentation of the scenarios in an online survey to facilitate understanding of the task [34].

## Footnotes

1 See https://www.excelc.eu/.

2 The ASCOT measure is disclosed in full herein but ordinarily should not be used for any purposes without the appropriate permissions of the ASCOT team and the copyright holder—the University of Kent. Please visit http://www.pssru.ac.uk/ascot or email ascot@kent.ac.uk to enquire about permissions.

3 Measurement effects are largely identified in studies with the same sampling frame whereby the same respondents provide different answers to different administration methods [7].

4 These questions related to (a) whether the respondent understood what he/she was being asked to do in the BWS task; (b) the amount of thought the respondent put into responding to the task; and (c) the degree of fatigue shown by the respondent during the task.

5 A/B: higher managerial/professional/administrative, intermediate managerial/professional/administrative; C1: supervisory or clerical/junior managerial/professional/administrative, student; C2: skilled manual worker; D/E: semi or unskilled manual work, casual worker—not in permanent employment, housewife/homemaker, retired and living on state pension, unemployed or not working due to long-term sickness, full-time carer of other household member.

6 The results from the test statistic indicated that the null hypothesis of homogeneity in preferences across the two datasets was rejected at 1% significance level.

7 The separate models were used as a base in this additional pooled model. The results from the separate models are available upon request.

8 We tested for gender, age, current quality of life, experience with long-term care needs, religion, education, marital status, living area, current employment status, income, number of adults in the household, tenure, core benefits, disability benefits and ASCOT items.

9 Failure to account for the repeated nature of the data would potentially lead to downward biased standard errors [15].

10 IQR: Interquartile range.
